# Climatic Parameters and Rotavirus Diarrhea Among Hospitalized Children: A Study of Eastern India

**DOI:** 10.3389/fped.2020.573448

**Published:** 2020-10-30

**Authors:** Vishwanath Ghoshal, Rashmi Ranjan Das, Manas Kumar Nayak, Snigdha Singh, Palash Das, Nirmal Kumar Mohakud

**Affiliations:** ^1^Department of Pediatrics and Community Medicine, Kalinga Institute of Medical Sciences (KIMS), Bhubaneswar, India; ^2^Department of Pediatrics, All India Institute of Medical Sciences (AIIMS), Bhubaneswar, India

**Keywords:** rotavirus diarrhea, under five, relative humidity, temperature, rainfall, acute gastroenteritis

## Abstract

**Background:** Rotavirus diarrhea is often referred as “winter diarrheal disease” as it causes nearly 50% of the pediatric hospitalizations during winter season. This study was done with the objective of bringing out the epidemiological nexus of rotavirus cases with different seasonal parameters like maximum, minimum temperature, humidity, and average rainfall.

**Methods:** This prospective observational study was conducted in a tertiary care teaching hospital of Eastern India from February 2016 to December 2018. Data on daily maximum and minimum temperature, relative humidity, and rainfall were collected.

**Result:** Of 964 children admitted, 768 stool samples were collected for rotavirus assay. A total of 222 children (29%) were positive. The maximum, minimum temperature, average rainfall, and average humidity of 83.4 mm, 79.2%, 28.1, and 21.9, respectively, were significantly associated with positive rotaviral cases.

**Conclusions:** The incidence of rotavirus positivity cases was found to be inversely associated with average temperature, humidity, and rainfall. The knowledge about the seasonal pattern in a particular geographical area would help in the reallocation of hospital services (staff and bed) to tackle the epidemic or emergency situations resulting from clustering of cases.

## Introduction

Diarrhea is a prime public health problem among the under-five age group in low- and middle-income countries. Globally, nearly 215,000 deaths due to rotavirus gastroenteritis have occurred in this age group, with four countries (Nigeria, Pakistan, India, and the Democratic Republic of the Congo) accounting for 49% of the total deaths (1). In India, before the introduction of rotavirus vaccine in 2016, nearly 30.6% of gastroenteritis cases requiring hospitalization in children under-five is due to rotavirus (2). It is obvious that diarrheal diseases are prevailing in low-income countries that have poor access to uncontaminated water, sanitation, and streamlined medical care (1, 2). This disease is also a recurrent cause of hospitalization in the high-income countries—making diarrhea an important health concern globally. Rotavirus is the most common viral cause of diarrhea among children under-five in developing countries often referred as “winter diarrheal disease,” because it is responsible for 50% of the pediatric hospitalizations during winter season (3, 4).

Seasonal pattern plays an important role in diarrheal diseases. Bacterial diarrhea occurs frequently during the warm season in temperate climates, whereas diarrhea caused by viruses, particularly the ones by rotavirus, peaks in the cold weather (5). In tropical climates, rotavirus diarrhea occurs throughout the year (increasing its frequency during the dry and cold months), whereas bacterial diarrhea peaks in warm and rainy seasons (6). The incidence of persistent diarrhea follows the seasonal pattern of acute watery diarrhea.

The data show that every 1°C increase in temperature is associated with a 4–10% decrease in the incidence of rotavirus-associated diarrheal disease in the tropics (7, 8). In the temperate zone, it is discerned that rotavirus is frequent during the cool weather months (9). Numerous studies have assessed fomite transmission, and though rotavirus can survive on fomites for a long period of time (in the order of weeks), there is a lack of evidence to support that temperature has a strong association with rotavirus's persistence on fomites (7, 8, 10). In the tropical zone, the low- and middle-income countries may have an alternative environmental pathway (e.g., water) that can contribute to the temperature-driven incidence pattern of retrovirus infection. Seasonality indirect transmission of rotavirus in the temperate zone is conceivably explained by the large seasonal shifts (7, 8). Also, rainfall and humidity have an effect on rotaviral cases, shown that level of rainfall and humidity is inversely related to the number of rotaviral positive cases (11, 12).

Documentation of seasonal/climatic parameters on diarrheal disease epidemiology in the Eastern part of India is meager. Odisha, an eastern Indian state, has a climate that follows four-season pattern. In the present study, we tried to explore the epidemiological nexus of rotavirus cases with different seasonal parameters like maximum, minimum temperature, humidity, and average rainfall.

## Materials and Methods

This prospective observational study was conducted in the pediatrics department of a tertiary care teaching hospital (Kalinga Institute of Medical Sciences (KIMS) Bhubaneswar) of Eastern India from February 2016 to December 2018. Stool samples were collected from hospitalized children after enrollment into the study. After collection, stool samples were kept at −70°C temperature. Subsequently, they were placed in vaccine carriers with ice packs and transported to the referral laboratory at the Christian Medical College (CMC) Vellore once every month. Institutional ethical committee approval was obtained from KIMS Bhubanswar. Written, informed consents were obtained from the parents or guardians of enrolled children.

### Laboratory Procedure

After being received at CMC Vellore, the stool samples were kept at −70°C temperature till further testing. The samples initially underwent screening for rotavirus VP6 antigen by enzyme immunoassay (EIA; Premier™ Rotaclone, Meridian Biosciences Inc., Cincinnati, USA) (6). Manufacturer's instructions were followed in conducting the assay. As recommended by the manufacturer, positive reporting was done for samples with an OD value of ≥0.150. For samples positive by EIA, genotyping PCR was performed to determine the RV genotype. Automated method (QIAGEN, QIAcube HT) was used for extraction of viral RNA from 20% (*W*/*V*) stool suspension (0.2 g stool in 1 mL MEM). By modus operandi of reverse transcription, the RNA of virus was converted to complementary DNA (cDNA). It was done with “Invitrogen” —random primer and 200 U/μL of Moloney murine leukemia virus reverse transcriptase enzyme (Superscript II MMLV-RT, Invitrogen). For genotyping, the cDNA was used as template in a hemi-nested multiplex PCR for detecting genes—VP7 (G type) and VP4 (P type), using the published oligonucleotide primers (13–16). In the PCR, primers to identify G1, G2, G3, G4, G8, G9, G10, and G12 (VP7 genotypes) and P4, P6, P8, P9, P10, and P11 (VP4 genotypes) were included. Identification of genotypes was based on diversified band sizes of the products. For samples reported negative by PCR genotyping, a VP6 conventional PCR was executed to obviate rotavirus positivity.

Data on daily maximum and minimum temperature, relative humidity, and rainfall were collected with the help of Accuweather Internet source and Website in Odisha state (in the Eastern part of India) meteorological data (17, 18). The computation of daily average temperature was done as the mean of the daily maximum and minimum values. The monthly mean for average temperature, relative humidity, and total rainfall measured were obtained and calculated from the daily records. Seasons were categorized as March–May (pre-monsoon), June–September (monsoon), October–November (post-monsoon), and December–February (winter) (11).

### Statistical Analysis

The data were analyzed by STATA software (version 20). The independent *t* test was used to analyze the results among rotavirus-positive and rotavirus-negative cases obtained with the parameters of average rainfall, humidity, and average maximum and minimum temperature of the past 3rd day from the date of admission after taking into consideration, 2-day incubation period for rotavirus disease.

## Results

Of 964 children under-five hospitalized with acute gastroenteritis (AGE) during the study period, 768 (80%) provided stool samples for rotavirus assay. A total of 222 children (29%) were positive. The year-wise average monthly enrollment and positivity of children was as follows: 2016 (enrolled = 16; positive = 6), 2017 (enrolled = 24; positive = 5), and 2018 (enrolled = 23; positive = 7). Of the 768 cases, males dominated over females (53 vs. 47%), and the most common age group affected was <12 months (38%). Cases from the rural area dominated over the urban (51 vs. 49%). The most common source of drinking water was the one shared by the community (40%), and many cases belong to the lower to middle socioeconomic status (61%). The association of positive and negative results with the parameters of average rainfall, average humidity, and maximum and minimum temperature prevailing in the previous 3rd day from the date of hospitalization is provided in Table 1. The average rainfall for rotaviral-positive cases was 83.4 and 123.9 mm for negative cases. The average humidity for rotaviral-positive cases was 79.2 ± 16.1% and 82.7 ± 12.7% for negative cases. The average minimum atmospheric temperature for rotaviral positive cases was 21.9 ± 4.8°C and 24.2 ± 4.6°C for negative cases. The average maximum temperature for rotaviral-positive cases was 28.1 ± 6.2°C and 29.0 ± 5.6°C for negative cases. All the differences were statistically significant between the rotaviral-positive and rotaviral-negative groups (*p* < 0.001).

**Table 1:** Association of different climatic parameters with positive and negative rotavirus diarrheal cases.

**Parameters**	**Results**		***P*-value**
	**Positive cases** **(*n* = 222)**	**Negative cases** **(*n* = 546)**	
Average rainfall	83.4 ± 171.5 mm	123.9 ± 162.4 mm	<0.001
Average humidity	79.2 ± 16.1%	82.7 ± 12.7%	<0.001
Maximum temperature	28.1 ± 6.2°C	29.0 ± 5.6°C	<0.001
Minimum temperature	21.9 ± 4.8°C	24.2 ± 4.6°C	<0.001

**Figure 1:**
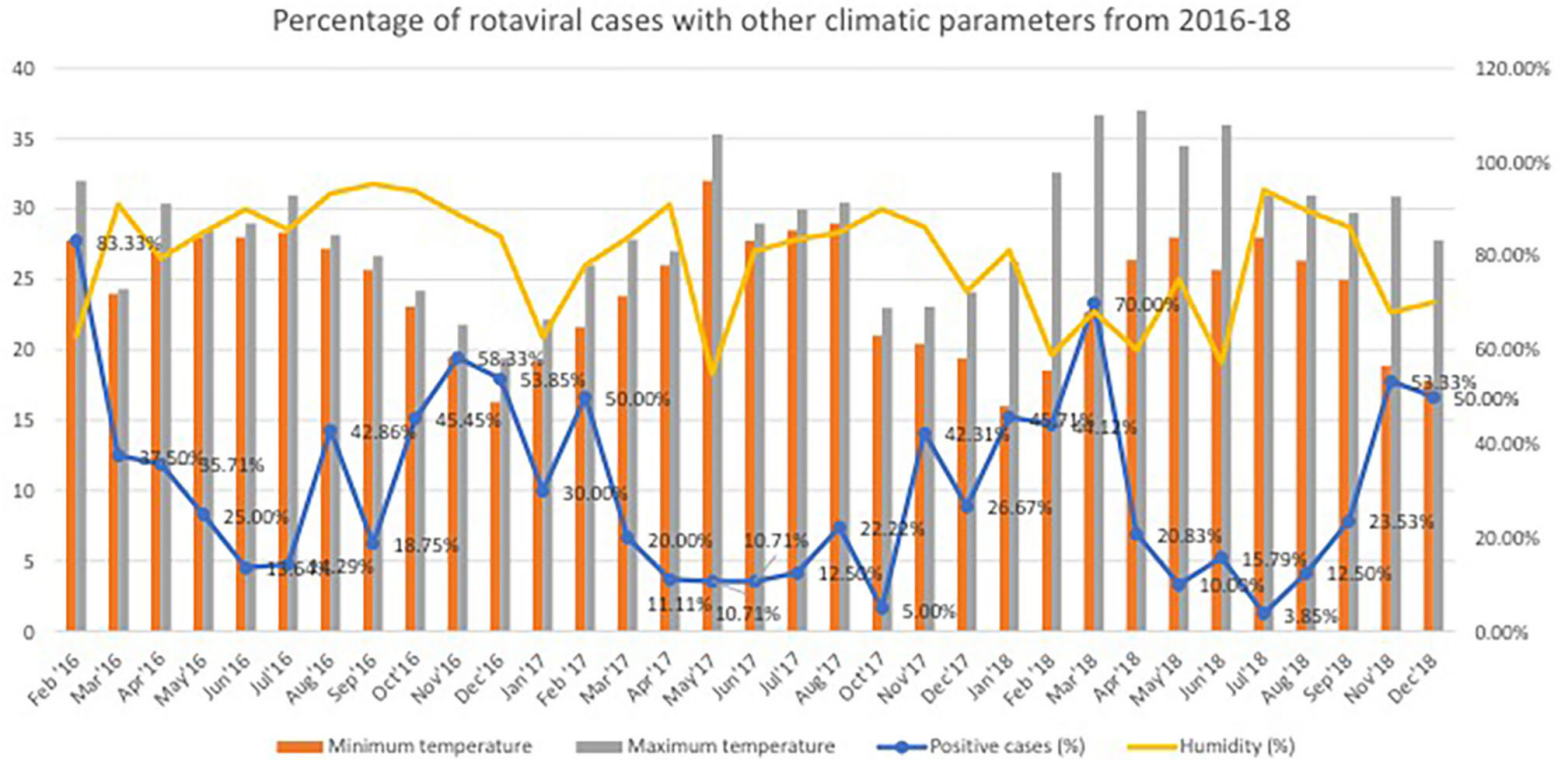
Percentage of rotaviral cases with other climatic parameters from 2016 to 2018.

Figure 1 provides a graphical representation of different climatic parameters of minimum and maximum temperature, humidity, and the percentage of positive rotavirus diarrheal cases in the years 2016, 2017, and 2018, respectively. During the start of the study in February 2016, we observed an increase percentage of rotaviral-positive cases (about 80% of total admitted diarrheal cases). The percentage of cases climbed to >50% again in the month of November and December 2016. This shows that the cases are mostly confined to winter months of the year. Similarly, the peaks were recorded during February and November 2017. The winter months showed an increase percentage of positive cases, with nearly 30–50% positive cases occurring in this season. We found 50% cases in almost all the winter months with a steep increase (70%) of positive cases in March 2018.

**Figure 2:**
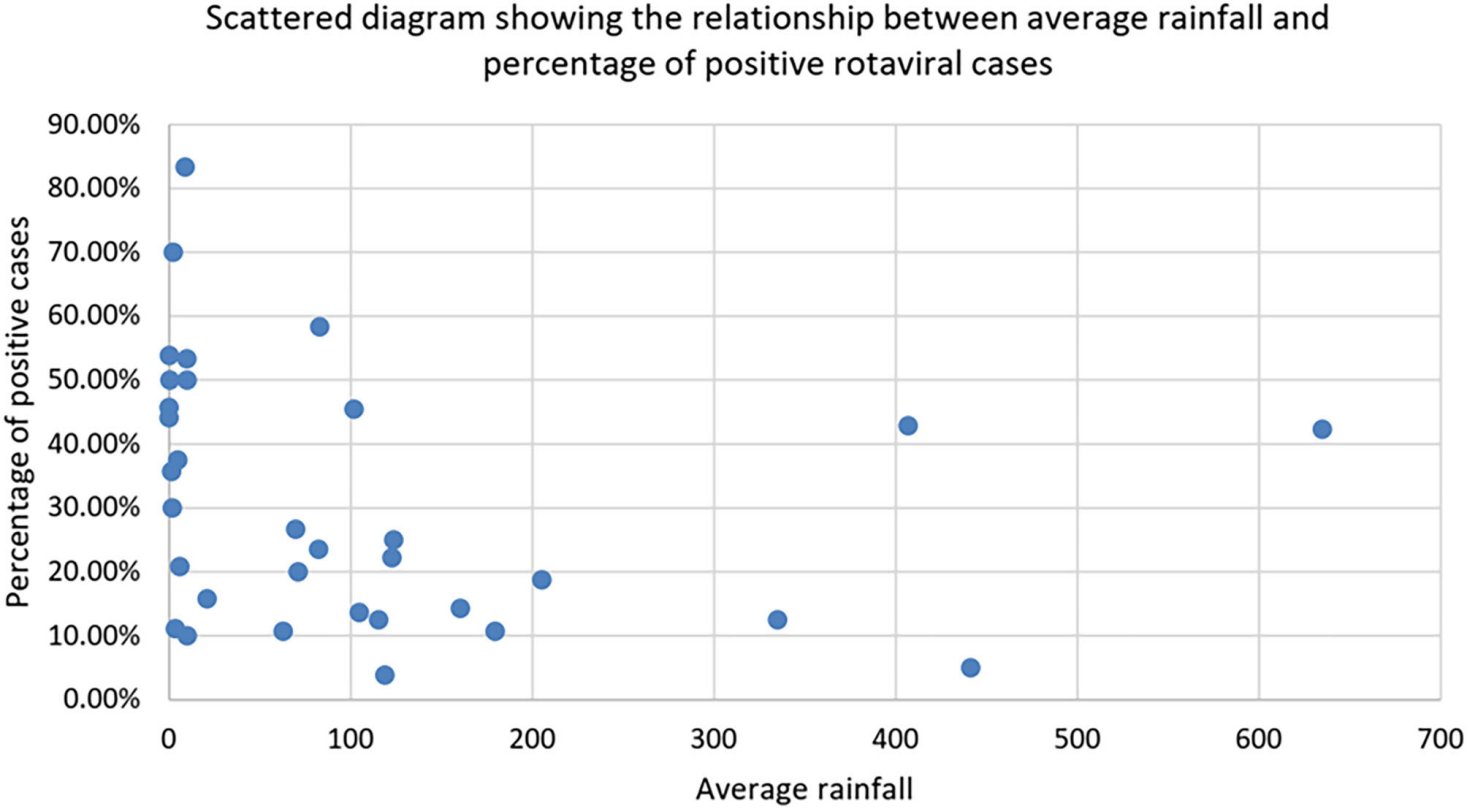
Scattered diagram showing the relationship between average rainfall and percentage of positive rotaviral cases.

Figure 2 clearly shows that positive rotaviral cases are mostly confined to the area with an average rainfall of <100 mm. There is also an inverse relationship between the percentage of rotaviral cases and average rainfall. As the rainfall rises to >100 mm, the positive case percentage was decreased with very few positive cases in >200 mm.

**Figure 3:**
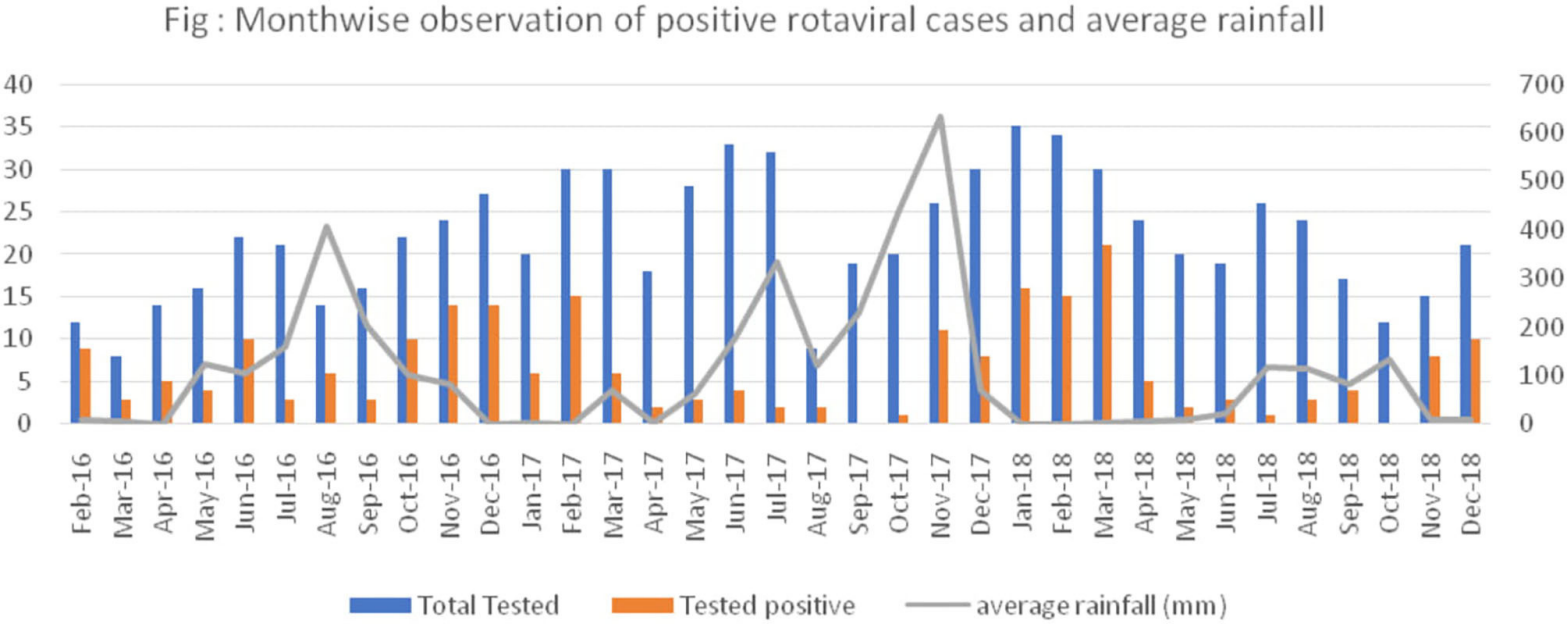
Month-wise observation of percentage of positive rotaviral cases and average rainfall.

As shown in Figure 3, by plotting a graph between the percentage of rotaviral cases and average rainfall, we could observe an increase in the positive rotaviral cases with a decrease in average rainfall (from February to April 2016, October to March 2017, and January to March 2018). Other parameters might have played an important role, which could be a reason for low rotaviral cases in some months despite a lower range of average rainfall.

Besides the above findings, the present study reported P8 and G3 genotypes as the most prevalent among the other genotypes with a frequency of 81.90 and 52.94%, respectively. The G3 genotype in combination with other genotypes (G1, G9, G10, and G12) was also evident with a cumulative percentage of 14.5%. Furthermore, P8 was also reported in combination with P4 in 2% cases.

## Discussion

The present study found a significant association of rotavirus diarrheal cases with the seasonal parameters. As the average humidity, temperature, and rainfall increased, rotavirus (RV) positivity rate decreased, leading to a decrease in hospital admissions.

Humidity of 60–79% were found to be favorable for rotaviral disease as per present study, while >83% did not favor it (except for some exceptions in 2018), which could be due to other parameters like maximum and minimum temperature. When all the climatic parameters like relative humidity, average rainfall, and maximum and minimum temperature are studied simultaneously, it is difficult to predict which parameter is actually influencing rotavirus transmission. The transmission of rotavirus over a vast geographic area during the winter in the temperate zones reports that water alone may not be responsible for all RV spread (19).

One study found that with every 1°C rise in temperature, an increase in the average rainfall of 1 cm, and a 1% increase in the relative humidity, there was a reduction in the rotavirus case incidence by 10, 1, and 3%, respectively (7). Another study done in Australia had found higher temperature and humidity to be associated with increase in rotaviral diarrhea admissions (9). The authors also found the admissions to peak in winter and spring seasons while it was lowest in summer. The present study has a similar observation with peaks during the winter; however, in addition, there was also a rise in the cases in the post-monsoon season (i.e., October and November). In temperate climate, rotaviral diarrhea occurs mainly during the fall and winter; but in tropical climate and developing countries, this seasonality is marked less (3, 19–21). Unlike other countries of the temperate zone, the tropical climate of India favors rotaviral cases throughout the year (11). In a study from Kolkata which is very close to the present study city in India, the observations were similar (11). The authors of this study also found an inverse association of rotavirus-positive cases with that of temperature, humidity, and rainfall (11). The peaks in cases of rotavirus cases during the cold season have also been found in other geographical areas of India (22–26). This is secondsary to both outdoor and indoor factors (4, 7, 9). The outdoor factor includes prolonged survival of the virus outside and the indoor factor involves people getting confined to their home during winter or cold season, thereby having a higher risk of exposure to index cases. The present study found a rise in the incidence of positive cases during the months of June and July. The number of cases decreased abruptly in the month of August, during which, there is maximum annual rainfall along with towering humidity of 90–100%. Similar was the finding from previous studies (7, 11). In the tropical climate, increase in temperature is associated with an increase in rainfall and humidity. So, it may happen that increase in temperature, rainfall, and humidity goes hand in hand to exert the same effect on rotavirus diarrhea cases (11).

Thus, we find that unlike temperate zone, rotavirus transmission occurs almost throughout the year in tropical countries like India. Although in summer months it may decrease, its existence in the environment is inevitable. For its reappearance at the beginning of the next season, it requires either the introduction of a new strain from outside or the re-emergence of the old strains from some yet to be identified reservoir in the environment that gets facilitated by the environmental factors (e.g., an increase in rainfall and humidity).

The present study has some limitations. Firstly, being a hospital-based data, there is a chance of selection bias, as the cases are likely to be different from those in the community. Secondly, we assume that the cases were mostly either moderate or severe as mild case might have gone unreported because of hospital-based data. Thirdly, we did not take into account or control the role of other factors (e.g., dietary habits, socioeconomic factors, housing status, contact with other cases, indoor humidity, and temperature) that have the potential to affect the reported rotaviral cases.

## Conclusions

There is a positive association between maximum, minimum temperature, average rainfall, and average humidity with a percentage prevalence of rotaviral cases. The knowledge about the seasonal pattern in a particular geographical area would help in the reallocation of hospital services (staff and bed) to tackle the epidemic or emergency situations resulting from clustering of cases.

## Data Availability Statement

The raw data supporting the conclusions of this article will be made available by the authors, without undue reservation.

## Ethics Statement

The studies involving human participants were reviewed and approved by Institutional Ethics Committee KIMS, KIIT Deemed to be University. Written informed consent to participate in this study was provided by the participants' legal guardian/next of kin.

## Author Contributions

NM: concept, design, patient management, collecting data, writing the draft, and act as guarantor of the paper. VG, SS, PD, and MN: patient management, collecting data, and writing the draft. RD: concept, performing statistical analysis and making inferences, and writing the draft. All the authors have approved the version to be published.

## Conflict of Interest

The authors declare that the research was conducted in the absence of any commercial or financial relationships that could be construed as a potential conflict of interest.
